# Complete plastome sequences of *Diospyros maclurei* Merr. and *Diospyros hainanensis* Merr. (Ebenaceae): two endemic species in Hainan Province, China

**DOI:** 10.1080/23802359.2018.1524724

**Published:** 2018-10-30

**Authors:** Wen-Wen Liu, Xin-Hang Tan, Kun-Kun Zhao, Zhi-Xin Zhu, Hua-Feng Wang

**Affiliations:** Hainan Key Laboratory for Sustainable Utilization of Tropical Bioresources, Institute of Tropical Agriculture and Forestry, Hainan University, Haikou 570228, China

**Keywords:** *Diospyros maclurei* Merr., *Diospyros hainanensis* Merr., plastome, phylogeny, genome structure, Ebenaceae

## Abstract

*Diospyros* is the largest genus (about 485 species) in Ebenaceae. It is a deciduous or evergreen tree or shrub. It grows in pantropical and extending into temperate regions. Here, we report and characterize the complete plastid genome sequences of *D. maclurei* Merr. and *D. hainanensis* Merr. in an effort to provide genomic resources useful for promoting their systematics research and potential economic development. The complete plastome of *D. maclurei* is 157,946 bp in length, including two Inverted Repeat (IR) regions of 26,081 bp, a Large Single-Copy (LSC) region of 87,387 bp, and a Small Single-Copy (SSC) region of 18,397 bp. The plastome contains 114 genes, consisting of 80 unique protein-coding genes, 30 unique tRNA genes, and four unique rRNA genes. The overall A/T content in the plastome of *D. maclurei* is 62.60%. The complete plastome of *D. hainanensis* is 157,999 bp in length, including two Inverted Repeat (IR) regions of 26,077 bp, a Large Single-Copy (LSC) region of 87,523 bp, and a Small Single-Copy (SSC) region of 18,322 bp. The plastome contains 114 genes, consisting of 80 unique protein-coding genes, 30 unique tRNA genes, and four unique rRNA genes. The overall A/T content in the plastome of *D. hainanensis* is 62.60%. The complete plastome sequences of *D. Maclurei* and *D. hainanensis* will provide a useful resource for the conservation genetics of the two species as well as for the phylogenetic studies of *Diospyros.*

## Introduction

*Diospyros maclurei and Diospyros hainanensis* were sampled from Diaoluo Mountain (109.88°E, 18.67°N), which is a National Nature Reserve of Hainan, China. The voucher specimens (*D. maclurei*, Wang et al., B51 and *D. hainanensis*, Wang et al., B10) were deposited in the Herbarium of the Institute of Tropical Agriculture and Forestry (HUTB), Hainan University, Haikou, China. The experiment procedure is as reported in Zhu et al. (2018). Around 6 Gb clean data were assembled against the plastome of *D. blancoi* (NC033502.1) (Jo et al. 2016) using MITObim v1.8 (Hahn et al. 2013). The plastome was annotated using Geneious R8.0.2 (Biomatters Ltd., Auckland, New Zealand) against the plastome of *D. blancoi* (NC033502.1) (Jo et al. 2016). The annotation was corrected with DOGMA (Wyman et al. 2004). The plastomes of *D. maclurei* and *D. hainanensis* were found to possess a total length 157,946 bp and 157,999 bp, respectively. They both have typical quadripartite structure of angiosperms, containing two Inverted Repeats (IRs) of 26,081 bp in *D. maclurei* and 26,077 bp in *D. hainanensis*, a Large Single-Copy (LSC) region of 87,387 bp (*D. maclurei*) and 87,523 bp (*D. hainanensis*), and a Small Single-Copy (SSC) region of 18,397 bp (*D. maclurei*) and 18,322 bp (*D. hainanensis*). Both plastomes contain 114 genes, consisting of 80 unique protein-coding genes (six of which are duplicated in the IR), 30 unique tRNA gene (seven of which are duplicated in the IR) and four unique rRNA genes (16S rRNA, 23S rRNA, 4.5S rRNA, and 5S rRNA). Among these genes, two pseudogenes [*ycf1* (translation from 112,295 to 113,481) and *ycf1* (translation from 133,039 to 127,419)] in *D. maclurei* and one pseudogene [*ycf1* (translation from 112,427 to 113,613)] in *D. hainanensis*, 15 genes possessed a single intron and three genes (*ycf3, clpP, rps12*) had two introns. The gene *rps12* was found to be trans-spliced, as is typical of angiosperms. The overall A/T content in the plastome of *D. maclurei and D. hainanensis* is 62.60%, which the corresponding value of the LSC, SSC, and IR region were 64.60%, 69.20%, and 56.90%, respectively.

**Figure 1.**
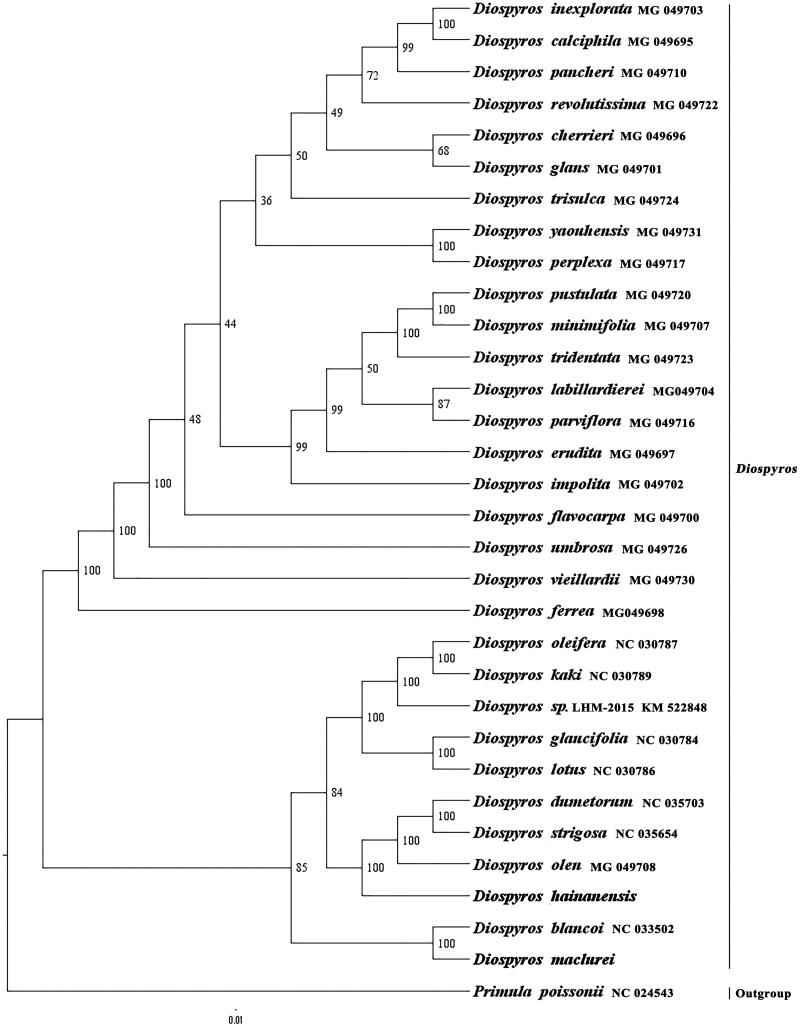
The best ML phylogeny recovered from 32 complete plastome sequences by RAxML. Accession numbers: *Diospyros maclurei* Merr. (This study, GenBank accession number: MH778101), *Diospyros hainanensis* Merr. (This study, GenBank accession number: MH778100), *Diospyros minimifolia* MG_049707, *Diospyros pustulata* MG_049720, *Diospyros tridentata* MG_049723, *Diospyros labillardierei* MG_049704, *Diospyros parviflora* MG_049716, *Diospyros erudita* MG_049697, *Diospyros impolita* MG_049702, *Diospyros perplexa* MG_049717, *Diospyros yaouhensis* MG_049731, *Diospyros flavocarpa* MG_049700, *Diospyros umbrosa* MG_049726, *Diospyros calciphila* MG_049695, *Diospyros inexplorata* MG_049703, *Diospyros pancheri* MG_049710, *Diospyros revolutissima* MG_049722, *Diospyros glans* MG_049701, *Diospyros cherrieri* MG_049696, *Diospyros trisulca* MG_049724, *Diospyros vieillardii* MG_049730, *Diospyros ferrea* MG_049698, *Diospyros oleifera* NC_030787, *Diospyros kaki* NC_ 030789, *Diospyros sp.* LHM-2015 KM_522848, *Diospyros glaucifolia* NC_ 030784, *Diospyros lotus* NC _030786, *Diospyros dumetorum* NC_ 035703, *Diospyros strigosa* NC_ 035654, *Diospyros olen* MG_049708, *Diospyros blancoi* NC _033502; outgroup: *Primula poissonii* NC_024543.

We used RAxML (Stamatakis, 2006) with 1000 bootstraps under the GTRGAMMAI substitution model to reconstruct a maximum likelihood (ML) phylogeny of 29 published complete plastomes of Ebenaceae, using *Primula poissonii* (Sapotaceae) as outgroup. The phylogenetic analysis indicated that *D. maclurei* is close to *D. blancoi*, and *D. hainanensis* is close to the ancestor of *D. olen, D. dumetorum*, and *D. strigosa* within *Diospyros* (Figure 1). Most nodes in the plastome ML trees were strongly supported. The complete plastome sequences of *D. maclurei* and *D. hainanensis* will provide a useful resource for the systematics and economic development of the two species as well as for the phylogenetic studies of *Diospyros*.
